# Male Sex Is Associated with Higher Mortality and Increased Risk for Complications after Surgical Treatment of Proximal Humeral Fractures

**DOI:** 10.3390/jcm10112500

**Published:** 2021-06-05

**Authors:** Jeanette Koeppe, J. Christoph Katthagen, Robert Rischen, Moritz Freistuehler, Andreas Faldum, Michael J. Raschke, Josef Stolberg-Stolberg

**Affiliations:** 1 Institute of Biostatistics and Clinical Research, University of Muenster, Schmeddingstrasse 56, 48149 Muenster, Germany; Jeanette.Koeppe@ukmuenster.de (J.K.); Andreas.Faldum@ukmuenster.de (A.F.); 2 Department of Trauma-, Hand- and Reconstructive Surgery, University Hospital Muenster, Albert-Schweitzer-Campus 1, Building W1, 48149 Muenster, Germany; Christoph.Katthagen@ukmuenster.de (J.C.K.); michael.raschke@ukmuenster.de (M.J.R.); 3 Clinic for Radiology, University Hospital Muenster, Albert-Schweitzer-Campus 1, Building A1, 48149 Muenster, Germany; Robert.Rischen@ukmuenster.de; 4 Medical Management Division—Medical Controlling, University Hospital Muenster, Niels-Stensen-Straße 8, 48149 Muenster, Germany; Moritz.Freistuehler@ukmuenster.de

**Keywords:** proximal humeral fracture, locked plate fixation, reverse total shoulder arthroplasty, geriatric surgery, complications rates, sex differences, risk analysis, real world analysis, multivariable Cox regression

## Abstract

Aims: The best surgical treatment of multi-fragmentary proximal humeral fractures in the elderly is a highly controversial topic. The aim of this study is to assess for sex-related differences regarding mortality and complications after reverse total shoulder arthroplasty (RTSA) and locking plate fixation (LPF). Patients and Methods: All patients from the largest German healthcare insurance (26.5 million policy holders) above the age of 65 years that were treated with LPF or RTSA after a multi-fragmentary proximal humerus fracture between January 2010 and September 2018 were included. Multivariable Cox regression models were used to assess the association of sex with overall survival, major adverse events and surgical complications. Results: A total of 8264 (15%) men and 45,707 (85%) women were followed up for a median time of 52 months. After 8 years, male patients showed significantly higher rates for death (65.8%; 95% CI 63.9–67.5% vs. 51.1%; 95% CI 50.3–51.9%; *p* < 0.001) and major adverse events (75.5%; 95% CI 73.8–77.1% vs. 61.7%; 95% CI 60.9–62.5%; *p* < 0.001). With regard to surgical complications, after adjustment of patient risk profiles, there were no differences between females and males after LPF (*p* > 0.05), whereas men showed a significantly increased risk after RTSA (HR 1.86; 95% CI 1.56–2.22; *p* < 0.001) with more revision surgeries performed (HR 1.76, 95% CI 1.46–2.12; *p* < 0.001) compared to women. Conclusion: The male sex is an independent risk factor for death and major adverse events after both LPF and RTSA. An increased risk for surgical complications after RTSA suggests that male patients benefit more from LPF. Sex should be considered before making treatment decisions.

## 1. Introduction

The proximal humeral fracture is the third most common fracture in patients older than 65 years of age and represents 5.3% of all fractures with an incidence of 25.3/10,000 [1,2,3]. During the next fews years, a dramatic increase in incidence is predicted due to demographic changes [4,5]. In particular, female patients are affected with an exponential increase in risk from the age of 40 onwards [3,6]. Furthermore, complex proximal humeral fractures occur more frequently in elderly women (1.72-fold), emphasizing the need for sex specific research [7,8].

Currently, open reduction and internal fixation using locked plate fixation (LPF) and reverse total shoulder arthroplasty (RTSA) are the most commonly performed and competing surgical procedures [5,9]. Within the past decade, there has been a strong increase in RTSA, reflecting high satisfaction and reliably good clinical results [10,11]. While first clinical studies confirm the superiority of RTSA over LPF [12,13,14], there is significant controversy amongst orthopedic surgeons on the optimal operative treatment strategy [15,16]. Subsequently, individualized treatment decisions are necessary to further improve outcomes.

Sex-related differences in proximal humeral fractures include bone quality and skeletal geometry, lower bone mineral density, reduced microarchitecture and loss of bone mass at the lateral region of the proximal humerus in female patients, reflecting osteoporotic characteristics [17,18]. In contrast to women, men suffering from a proximal humeral fracture are associated with excess mortality [19,20]. Despite sex-specific risk differences, adapted surgical strategies and analyses of perioperative risk factors are lacking in clinical practice.

We hypothesize that in elderly patients with proximal humeral fracture treated with LPF or RTSA, female patients show a significantly higher overall survival, less major adverse events and fewer surgical complications during follow-up.

## 2. Methods

### 2.1. Database and Patient Cohort

**Figure 1:**
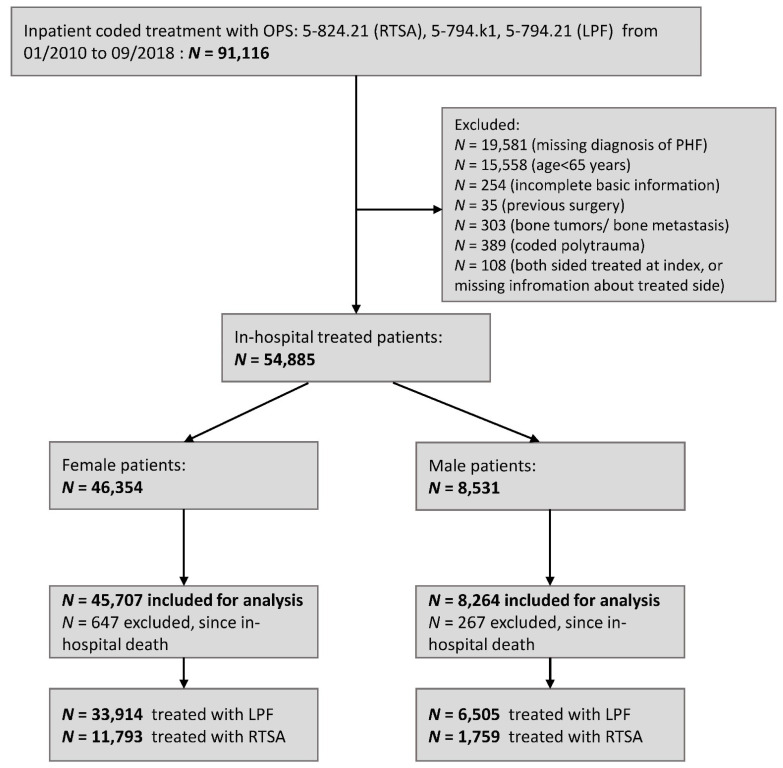
Flow diagram. Locked plate fixation—LPF, proximal humeral fracture—PHF, reverse total shoulder arthroplasty—RTSA.

In Germany, the healthcare system is dominated by mandatory health insurance and the use of inpatient and outpatient reimbursement systems. Settlement and coding are clearly defined by legal and statutory regulations, including encoded diagnoses (International Statistical Classification of Diseases, German Modification—ICD) and procedures (German procedure classification; Operations und Prozedurenschlüssel—OPS). Therefore, insurance data are characterized by their completeness and validity [21]. In this study, data from the Federal Association of the Local Health Insurance Funds (Allgemeine Ortskrankenkasse—AOK) were analyzed. The AOK is the largest statutory health insurance company in Germany with approximately 26.5 million insurance holders (representing approximately one-third of the German population). Retrospective patient data were available from January 2008 to December 2018. All patients with an age of 65 years and older at index hospitalization, treated with an inpatient LPF (OPS: 5-794.k1 or 5-794.21) or RTSA (OPS: 5-824.21) and an inpatient coded diagnosis of a multi-fragmentary PHF (ICD: S42.2) were included for further analysis within an index period from January 2010 to September 2018 (*n* = 53,971, see Figure 1). For a median time of 52 months, all patients were followed up from discharge of the initial hospitalization to the end of follow-up, defined as exit from the database, death or end of the study. Exclusion criteria were incomplete basic information (*n* = 254), incomplete information on index surgery (*n* = 108), coded polytrauma (*n* = 389), previous surgery with LPF or RTSA (*n* = 35), bone tumors/bone metastasis (*n* = 303) or death during index hospitalization (*n* = 941). Comorbidities at the index case and primary endpoints were determined according to primary and secondary diagnoses and procedures during index hospitalization, within inpatient and outpatient data from the previous 24 months and during the follow-up period, respectively (Supplementary Materials, Table S1). Moreover, the Charlson Comorbidity Index (CCI) was calculated at baseline, including all inpatient and outpatient coded diagnosis within the pre-phase [22,23,24]. The Anatomical Therapeutic Chemical classification system (ATC) was used to specify pharmacotherapy of any anticoagulant, vitamin D/calcium and bisphosphonates before index surgery.

### 2.2. Primary Endpoints

Regarding the primary research questions of this study, female and male sex in both treatment groups were compared with respect to:

- Major adverse events (MAE) (defined by resuscitation, cardiac arrest, myocardial infarction, stroke, acute renal failure, acute liver failure, acute respiratory distressed syndrome, sepsis or death from any case).
- Overall survival (OS) (defined by time to death from any case).
- Thromboembolic event or death (deep vein thrombosis, pulmonary embolism, ischemic stroke or death).
- Any surgical complications after discharge (adhesive capsulitis, arthrolysis, debridement, decompression of subacromial space, frozen shoulder, infection, infection with antibiotic-resistant germs, joint damage/cartilage damage, luxation, delayed union, non-union/pseudoarthrosis, malunion, nerve injury, vascular injury, osteonecrosis, postoperative stiffness, secondary arthroplasty, secondary arthroscopy, secondary surgery (open) including revision surgery, upper limb amputation (shoulder or upper arm)).
- Any surgical complications also including the index case.
- Any secondary ipsilateral surgery of the shoulder.
- Minor outpatient complications.

Detailed information on the definition and relating coding of all endpoints are presented in the Supplementary Materials, Table S1. 

### 2.3. Statistical Methods 

Differences between female and male sex in comorbidities and in-hospital outcome during the index case were tested via the χ^2^ test for categorical variables and two-sided Mann–Whitney U test for continuous variables. For all studied endpoints, the time from discharge of the index hospitalization to the target event were analyzed. Event rates at one year up to eight years after discharge from the initial hospitalization were calculated using Kaplan–Meier estimates for OS, MAE, thromboembolic events or death and differences between female and male sex were tested via a two-sided log-rank test for both treatment groups separately. The cumulative incidence function (event rate) at 1 to 8 years after discharge for all surgical complications were determined using the Nelson–Aalen estimator and tested via two-sided Gray’s tests to account for the competing risk of death. In order to account for inhomogeneities between women and men, multivariable Cox regression models including age, sex, treatment group, an interaction term sex*treatment group, year of surgery and patient’s risk profile were used to determine hazard ratios (HRs) for male sex (compared to female sex) depending on the treatment group for all primary endpoints adjusted by the patient’s risk profile. For all surgical endpoints, death was again considered as a competing event and the Fine and Gray’s sub-distributional HRs were calculated and presented. All *p*-values comparing differences of the event rates/cumulative incidence functions between the female and male sex and the *p*-values of the HRs for sex of all primary endpoints in both treatment groups were jointly adjusted using the Bonferroni–Holm method to control the family-wise error rate with respect to the multiple comparison problem. *p*-values were compared with the overall significance level of 5%. All other *p*-values, i.e., *p*-values comparing comorbidities at index, are fully explorative and were interpreted accordingly. Statistical analyses were performed using SAS software V9.4 (SAS Institute Inc., Cary, NC, USA) and R version 3.6.0 (26 April 2019, R Foundation for statistical computing, Vienna, Austria).

### 2.4. Demographics and Comorbidities

Within the index period, we identified *n* = 45,707 female patients, with 25.8% treated with RTSA and *n* = 8264 male patients with a slightly lower rate of RTSA (21.3%, *p* < 0.001). In general, female patients were older (median age (interquartile range (IQR)): female sex 79 (10) vs. male sex 76 (11), *p* < 0.001), had higher rates of osteoporosis diagnosis (female sex 40.2% vs. male sex 15.7%, *p* < 0.001) and osteoporosis pharmacotherapy at index (female sex 11.3% vs. male sex 4.6%, *p* < 0.001). However, male patients had a higher prevalence of comorbidities such as cancer, diabetes mellitus, coronary heart disease, hypertension and atherosclerosis and had higher rates of coded nicotine and alcohol abuse during pre-phase (all *p* < 0.001, see Supplementary Materials, Table S2). Even during the index hospitalization, women had fewer major medical and surgical complications, and thus, also a shorter length of stay (see Supplementary Materials, Table S2). 

## 3. Results

### 3.1. Overall Survival 

**Figure 2:**
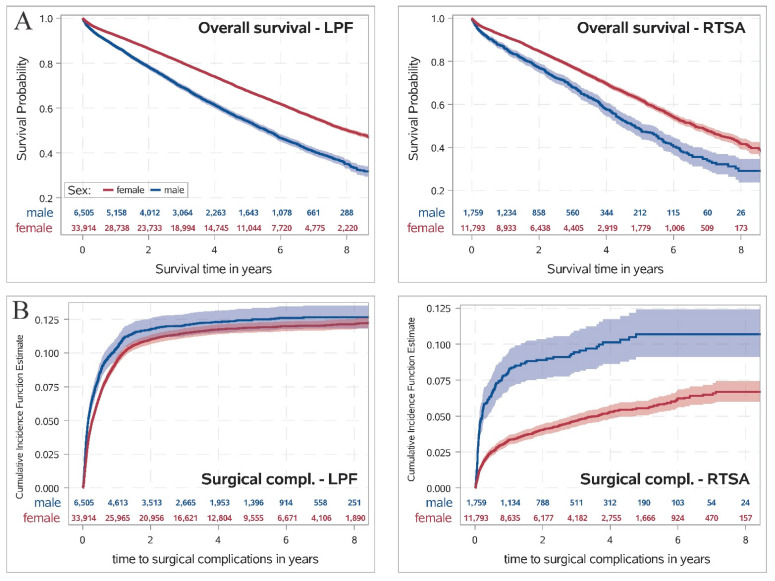
(**A**) Survival probability determined by Kaplan–Meier estimate for overall survival comparing female and male sex for both treatment groups. Differences between both sexes were tested using a two-sided log-rank test. (**B**) Cumulative incidence function determined by Nelson–Aalen estimate for surgical complications during follow-up for both treatment groups separately, where death was considered as a competing risk. Differences between female and male sex were tested using the two-sided Gray’s test. *p*-values were adjusted by the Bonferroni–Holm method. Locked plate fixation—LPF, reverse total shoulder arthroplasty—RTSA.

**Figure 3:**
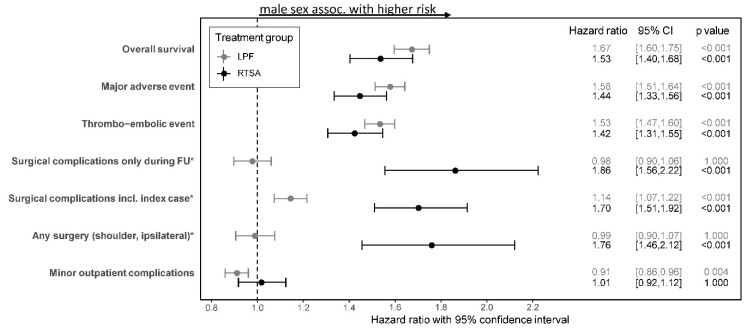
Association of sex with different target events during follow-up depending on treatment group determined by multivariable Cox regression models including sex, treatment group, interaction between sex and treatment group (sex * treatment group), age and patient’s risk profile at index surgery. *p* values were adjusted using Bonferroni–Holm procedures. For all studied events, the time from discharge of the index case to the target event was fitted. For any surgical complications, any surgery during follow-up, secondary ipsilateral surgery on the shoulder and minor outpatient complications, death was considered a competing risk and sub-distribution hazard ratios were estimated. Sub-distributional hazard ratios comparing male and female sex for the interaction of sex and treatment group, 95% CIs and adjusted *p* values are presented. Results of all regression models are collected in Supplementary Materials, Table S3. * significant differences of HR (comparing male vs. female sex) between treatment groups. CI—confidence interval, locked plate fixation—LPF, reverse total shoulder arthroplasty—RTSA.

In both treatment groups, female patients had a significantly higher overall survival compared to men (see Figure 2A, *p* < 0.001). Eight years after discharge, 49.8% (95% CI 48.9–50.7%) of the female patients treated with LPF and 65.0% (95% CI 63.0–66.9%) of male patients treated with LPF were dead. In the RTSA group, the rate of patients who had died eight years after discharge was even more pronounced in both sexes, while men had a significantly higher rate of deaths than women (male sex 71.0% (95% CI 65.0–76.1%) vs. female sex 58.3% (95% CI 55.8–60.6%), *p* < 0.001; see Table 1). After adjustment for patient’s risk profile, male sex was also associated with a significantly decreased overall survival in both treatment groups (*p* < 0.001; see Figure 3) with no statistically noticeable difference in the influence of sex on OS between both treatment groups (*p*-value of interaction n.s.).

### 3.2. MAE and Thromboembolic Event

In both treatment groups, male patients had significant higher rates for MAEs and thromboembolic events (or death) up to eight years after discharge (all *p* < 0.001; see Table 1). After controlling for patient’s risk profile, male sex was associated with a significant higher risk for MAEs and thromboembolic events for both treatment groups (all *p* < 0.001; see Figure 3). However, no differences in the association of male sex on MAEs and thromboembolic events in between the treatment groups were found (all *p*-values of interaction n.s.).

### 3.3. Surgical Complications

The cumulative incidence function for surgical complications after discharge for both treatment groups depending on sex is presented in Figure 2B. In the LPF group, no differences in the rate of surgical complications after discharge between both sexes were observed (*p* = 0.34; see Table 1). However, in the RTSA group, male patients had significantly higher rates for surgical complications after discharge (after eight years: 10.7%, 95% CI 9.1–12.4%) compared to female patients (after eight years: 6.7%, 95% CI 6.0–7.4%); *p* < 0.001). After controlling for all comorbidities, no association between sex and surgical complications was detected for patients treated with LPF, whereas a significant influence of male sex could be found in the RTSA group (HR 1.86, 95% CI 1.56–2.22, *p* < 0.001). The difference in the association between sex and surgical complication in both treatment groups was found to be statistically notable (*p*-value of interaction < 0.001).

Similar characteristics were observed for secondary surgeries during follow-up (see Table 1 and Figure 3). While no risk differences between both sexes were observed in the LPF group, male sex was associated with a significantly higher risk for any secondary surgery during follow-up for patients treated with RTSA (see Figure 3; interaction *p* < 0.001).

Including the observed surgical complications during index hospitalization, a statistically notable difference in the association with sex occurred in both treatment groups (interaction *p* < 0.001). However, male sex was then associated with a significantly higher risk of surgical complications (including index case and follow-up) in both treatment groups (see Figure 3). Furthermore, it was found that in the LPF group, female sex had a higher risk for minor outpatient complications, while no statistically notable differences between the influence of sex on minor complications between both treatment groups were observed (interaction n.s.; see Figure 3). 

## 4. Discussion

The most important findings of this study were that male patients have a higher risk for MAEs, thromboembolic events, and death after LPF and RTSA with no differences between treatment groups. Furthermore, men show an increased risk for surgical complications and revision surgeries only after RTSA. Minor outpatient complications occur more frequently after LPF in female patients. 

Musculoskeletal disorders and injuries that are characterized by sexual dimorphism are not routinely considered by orthopedic surgeons when making treatment decisions [25]. This is surprising as sex differences in patient-related outcomes and functionality after orthopedic surgery are well described in the literature [26,27,28,29]. This observation might partly be caused by sex-specific postoperative morbidity and mortality [30,31]. As there is substantial controversy regarding the optimal surgical treatment strategy for proximal humeral fractures even amongst experienced shoulder surgeons, knowledge of specific risk profiles is critical during treatment decision-making.

The sex gap in life expectancy has biological and non-biological origins. The main difference, however, is attributed to non-biological, i.e., behavioral and socio-economic reasons, such as smoking, alcohol consumption and occupational risks, which show national variations [32]. For example, for the Russian federation a difference of 14 years between male and female inhabitants was reported, while for Sweden, the a difference was only 4 years [33]. During the period of analysis of the presented study, the difference in life expectancies in Germany ranged from 4.7 to 5.2 years [34]. With a median survival rate after fracture of 7.8 years for women and 5.5 years for men, the effect of sex also appears to exist in the present study. This is consistent with the literature showing an excess mortality in men after proximal humeral fracture [19,20,35]. Patients after surgical treatment using RTSA or LPF are associated with a lower risk of death, which is most likely biased by the pre-fracture comorbidities that led to the decision for surgery [36,37]. Hence, the increased mortality in men after adjustment for comorbidities reported in the present study is novel in the literature. For ethical reasons, the authors explicitly do not recommend depriving male patients of surgical treatment. For future economic assessment, however, it will be a key factor when analyzing cost effectiveness.

An understanding of sex dependent disease progression and treatment outcome provides the opportunity to improve the quality of individualized care [38]. There are several studies reporting that being a woman is a risk for longer length of hospital stay, increased opioid requirements, urinary tract infections, blood transfusions as well as non-home discharge. On the other hand, female sex protects from sepsis, cardiovascular or renal complications, e.g., after total knee arthroplasty [39,40,41,42]. Another exemplary and well-investigated fracture entity is the proximal femur. Similarly, sex specific differences in anatomy and biomechanics are identified in the literature [43,44]. The reduction of bone mass and architecture after the menopause contributes to a sex-dependent incidence ratio of 2:1 between men and women [45]. Similar to the present study, men are more likely to die after a hip fracture than women, which can be explained by more comorbidities and a greater percentage of undiagnosed disease [46]. While sex dependent post-operative functionality revealed inconsistent results, recent studies suggest that sex-adapted rehabilitation programmes might improve overall outcomes [47]. After RTSA for proximal humeral fracture, however, sex-specific literature is scarce. A database analysis by Ezuma et al. confirmed an increased risk for secondary surgery and any complication in male patients, although their cohort included only 175 men [48]. In a multicentre retrospective review of 898 patients, Gallinet et al. reported a rate of 12.5% overall complications with a 5% revision rate, without a sub-analysis of patient sex [49]. Cazeneuve et al. retrospectively followed up 2 male and 33 female patients with a rate of 23% complications (two complex regional pain syndrome, four dislocations, one deep infection, one aseptic loosening) for a mean follow up of 86 months, but without a sufficient cohort size to make conclusions on the effect of gender [50]. Russo et al. reported five periprosthetic fractures, one ulnar neuropathy and three postoperative hematomas and not a single MAE or revision surgery in 6 male and 44 female patients within a 6-month follow up, suggesting a highly specialized author, who treated the entire cohort [51]. There is some evidence on postoperative functionality, however, that men profit more than women from RTSA for the treatment of rotator cuff tears or osteoarthritis [52]. Furthermore, Brock et al. analyzed a cohort of 5499 female and 6949 male patients receiving RTSA without specification of the reason for surgery. They concluded that men have a decreased risk of urinary tract infection, blood transfusion, prolonged length of hospital stay and an increased risk for surgical site infection and prolonged operating time [53]. To summarize, the results of the present study fill the knowledge gap regarding male risk profiles after RTSA, emphasizing male sex as an independent risk factor for MAEs and surgical complications after RTSA. 

Despite an extensive body of research on locked plate fixation after proximal humeral fracture, few studies account for sex differences. In a study analyzing 42 cases of PHF after LPF using the deltoid splitting approach in elderly patients, the authors found no sex difference in terms of functionality [54]. Among the factors associated with loss of function after LPF, sex does not appear to have an effect on outcome [55]. A recent systematic review on screw perforations after LPF failed to show sex differences due to missing information on sex [56]. To the knowledge of the authors, there is only one study by Porschke and colleagues that directly compares complications between LPF and RTSA in a geriatric population after PHF [57]. The authors analyzed 31 patients after LPF, 14 after RTSA and 14 after hemiarthroplasty with a mean follow up of 2.7 years, and found significantly more surgical complications and revision surgeries after LPF. However, sex-related differences were not considered and with a revision rate of 29% after LPF, the data seems to be at the upper range compared to the literature [58]. While clinical outcome studies, showing superior functional results after RTSA in the elderly, describe more adverse events and revisions after LPF compared to RTSA, they fail to assess the effect of sex [12,13,14]. Hence, with the analysis of 53,971 geriatric PHF patients, the present study fills the knowledge gap on sex-dependent complication rates after LPF and RTSA and clearly shows that the male sex is a risk factor for increased mortality, MAEs, thromboembolic events and surgical complications after RTSA. For clinical practice, the question of superiority between LPF and RTSA in geriatric patients cannot simply be answered by only comparing postoperative functionality and complications, as groups are too heterogenous. However, the presented data are one first step in evaluating differences in the probability of death and complications between both sexes. Our data suggest that male patients might profit from intensified postoperative out-patient care. Future research needs to focus on sub-group analyses and risk profiling. Clinical guidelines have to include a more differentiated view of groups according to sex, age, comorbidities and risk factors. 

The database used in the study was given by routinely collected insurance data for reimbursement objectives and were not explicitly collected for research purposes. However, due to mandatory coding instructions, the ICD and OPS codes are very complete and dependable. Since the database does not include information about functional outcome, pain, quality of live or lifestyle changes during follow-up, the study was focused on severe endpoints, which are reliably coded. Furthermore, there are no information available about the underlying reason for treatment decisions, size of hospital or surgeon’s experience, which leads to a potential selection bias in the cohort. However, only treatment for multi-fragment fractures were included to reduce the inhomogeneity between both treatment groups.

## 5. Conclusions

In a geriatric population, the male sex is associated with a higher risk for MAEs, thromboembolic events and death after LPF and RTSA, with no differences between treatment groups. Men show an increased risk for surgical complications and revision surgeries only after RTSA. Hence, male sex is an independent risk factor for complications after surgical treatment of PHF. Within the male cohort, significantly decreased revision rates after LPF compared to RTSA suggest that male patients benefit more from LPF.

## Figures and Tables

**Table 1:** Cumulative incidence function/event rates of primary outcomes and 95% confidence intervals depending on sex and treatment group.

	All Patients *n* = 53,971 (100%)	Female Sex *n* = 45,707 (84.7%)	Male Sex *n* = 8264 (15.4%)	*p* Value
Death from any case LPF:				<0.001
1-year rate	8.4% (8.1–8.6%)	7.6% (7.3–7.8%)	12.6% (11.8–13.4%)	
2-year rate	14.8% (14.5–15.2%)	13.5% (13.1–13.9%)	21.7% (20.6–22.7%)	
5-year rate	34.5% (34.0–35.1%)	32.4% (31.8–33.0%)	45.9% (44.4–47.4%)	
8-year rate	52.2% (51.4–53.0%)	49.8% (48.9–50.7%)	65.0% (63.0–66.9%)	
Death from any case RTSA:				<0.001
1-year rate	9.1% (8.7–9.7%)	8.4% (7.9–8.9%)	14.2% (12.5–15.9%)	
2-year rate	16.2% (15.6–16.9%)	15.2% (14.5–15.9%)	23.1% (21.0–25.3%)	
5-year rate	39.4% (38.2–40.7%)	37.7% (36.3–39.0%)	51.6% (48.0–55.1%)	
8-year rate	59.9% (57.7–62.1%)	58.3% (55.8–60.6%)	71.0% (65.0–76.1%)	
Major adverse event LPF:				<0.001
1-year rate	12.4% (12.1–12.8%)	11.3% (11.0–11.7%)	18.1% (17.2–19.1%)	
2-year rate	21.3% (20.9–21.8%)	19.7% (19.2–20.1%)	30.1% (28.9–31.3%)	
5-year rate	44.7% (44.1–45.3%)	42.4% (41.7–43.0%)	57.1% (55.6–58.5%)	
8-year rate	62.9% (62.1–63.6%)	60.6% (59.7–61.4%)	74.8% (73.0–76.5%)	
Major adverse event RTSA:				<0.001
1-year rate	14.2% (13.6–14.8%)	13.2% (12.6–13.9%)	20.2% (18.3–22.2%)	
2-year rate	24.1% (23.3–24.9%)	22.9% (22.0–23.7%)	32.4% (30.0–34.8%)	
5-year rate	50.0% (48.7–51.2%)	48.0% (46.7–49.4%)	62.9% (59.4–66.2%)	
8-year rate	68.4% (66.4–70.4%)	66.7% (64.5–68.7%)	79.8% (73.9–84.4%)	
Thromboembolic event LPF:				<0.001
1-year rate	11.0% (10.7–11.3%)	10.2% (9.9–10.6%)	15.1% (14.2–16.0%)	
2-year rate	18.9% (18.5–19.3%)	17.6% (17.2–18.1%)	25.5% (24.4–26.6%)	
5-year rate	40.9% (40.3–41.5%)	38.8% (38.2–39.4%)	51.8% (50.3–53.3%)	
8-year rate	58.5% (57.8–59.3%)	56.5% (55.6–57.3%)	69.5% (67.6–71.3%)	
Thromboembolic event RTSA:				<0.001
1-year rate	12.2% (11.6–12.8%)	11.5% (10.9–12.1%)	16.8% (15.1–18.6%)	
2-year rate	20.9% (20.2–21.7%)	19.9% (19.1–20.7%)	27.8% (25.5–30.1%)	
5-year rate	45.9% (44.7–47.2%)	44.4% (43.0–45.7%)	56.7% (53.1–60.1%)	
8-year rate	64.6% (62.5–66.6%)	63.4% (61.1–65.6%)	72.6% (66.8–77.5%)	
Surgical complications only after discharge LPF:				0.34
1-year rate	9.5% (9.3–9.8%)	9.4% (9.1–9.7%)	10.4% (9.6–11.1%)	
2-year rate	11.1% (10.8–11.4%)	11.0% (10.7–11.3%)	11.8% (11.0–12.6%)	
5-year rate	12.0% (11.7–12.3%)	11.9% (11.5–12.2%)	12.5% (11.7–13.3%)	
8-year rate	12.2% (11.9–12.7%	12.1% (11.8–12.5%)	12.7% (11.8–13.5%)	
Surgical complications only after discharge RTSA:				<0.001
1-year rate	4.0% (3.6–4.3%)	3.3% (3.0–3.7%)	8.2% (6.9–9.5%)	
2-year rate	4.7% (4.3–5.1%)	4.0% (3.7–4.4%)	8.9% (7.6–10.3%)	
5-year rate	6.2% (5.7–6.7%)	5.5% (5.1–6.1%)	10.7% (9.1–12.4%)	
8-year rate	7.2% (6.6–7.9%)	6.7% (6.0–7.4%)	10.7% (9.1–12.4%)	
Surgical complications incl. index case LPF:				<0.001
1-year rate	16.1% (15.8–16.5%)	15.6% (15.2–15.91%)	19.1% (18.2–20.1%)	
2-year rate	17.6% (17.2–17.9%)	17.0% (16.6–17.4%)	20.3% (19.3–21.3%)	
5-year rate	18.3% (17.9–18.7%)	17.8% (17.4–18.2%)	20.9% (19.9–21.9%)	
8-year rate	18.5% (18.1–18.9%)	18.0% (17.6–18.5%)	21.0% (20.0–22.1%)	
Surgical complications incl. index case RTSA:				<0.001
1-year rate	12.1% (11.5–12.7%)	10.9% (10.4–11.5%)	19.8% (18.0–21.7%)	
2-year rate	12.7% (12.1–13.3%)	11.6% (11.0–12.2%)	20.4% (18.6–22.4%)	
5-year rate	14.0% (13.4–14.7%)	12.8% (12.2–13.5%)	22.0% (20.0–24.2%)	
8-year rate	14.9% (14.2–15.7%)	13.9% (13.1–14.7%)	22.0% (20.0–24.2%)	
Any secondary surgery LPF:				0.32
1-year rate	9.0% (8.7–9.3%)	8.9% (8.6–9.2%)	9.8% (9.1–10.5%)	
2-year rate	10.5% (10.2–10.8%)	10.3% (10.0–10.7%)	11.0% (10.3–11.8%)	
5-year rate	11.2% (10.9–11.5%)	11.1% (10.7–11.4%)	11.7% (10.9–12.6%)	
8-year rate	11.3% (11.0–11.7%)	11.2% (10.9–11.6%)	11.9% (11.1–12.7%)	
Any secondary surgery RTSA:				<0.001
1-year rate	3.6% (3.2–3.9%)	3.0% (2.7–3.3%)	7.3% (6.1–8.5%)	
2-year rate	4.3% (3.9–4.6%)	3.7% (3.4–4.1%)	7.9% (6.7–9.3%)	
5-year rate	5.8% (5.3–6.3%)	5.2% (4.8–5.7%)	9.4% (7.9–11.1%)	
8-year rate	6.8% (6.2–7.5%)	6.4% (5.7–7.2%)	9.4% (7.9–11.1%)	
Change to RTSA (only LPF):				0.31
1-year rate	2.7% (2.6–2.9%)	2.8% (2.6–3.0%)	2.6% (2.2–3.0%)	
2-year rate	3.4% (3.2–3.6%)	3.5% (3.3–3.7%)	3.0% (2.6–3.4%)	
5-year rate	3.7% (3.5–3.9%)	3.8% (3.6–4.0%)	3.2% (2.8–3.7%)	
8-year rate	3.8% (3.6–4.0%)	3.9% (3.7–4.2%)	3.3% (2.8–3.7%)	
Minor outpatient complications LPF:				<0.001
1-year rate	20.8% (20.4–21.2%)	20.9% (20.4–21.3%)	20.3% (19.3–21.3%)	
2-year rate	24.9% (24.5–25.3%)	25.2% (24.7–25.6%)	23.5% (22.5–24.6%)	
5-year rate	30.6% (30.1–31.1%)	31.1% (30.6–31.7%)	27.8% (26.7–29.0%)	
8-year rate	33.4% (32.9–34.0%)	34.1% (33.5–34.7%)	29.8% (28.5–31.1%)	
Minor outpatient complications RTSA:				1
1-year rate	21.9% (21.2–22.6%)	21.5% (20.8–22.3%)	24.0% (22.1–26.1%)	
2-year rate	25.8% (25.0–26.6%)	25.7% (24.9–26.5%)	26.6% (24.5–28.7%)	
5-year rate	31.7% (30.8–32.6%)	31.9% (30.9–32.9%)	30.4% (28.0–32.9%)	
8-year rate	34.0% (32.9–35.2%)	34.6% (33.3–35.9%)	30.8% (28.3–33.3%)	

## Data Availability

The authors confirm that the data utilized in this study cannot be made available in the manuscript, the supplemental files, or in a public repository due to German data protection laws (‘Bundesdatenschutzgesetz’, BDSG). Therefore, they are stored on a secure drive in the AOK Research Institute (WIdO), to facilitate replication of the results. Generally, access to data from statutory health insurance funds for research purposes is possible only under the conditions defined in German Social Law (SGB V § 287). Requests for data access can be sent as a formal proposal specifying the recipient and purpose of the data transfer to the appropriate data protection agency. Access to the data used in this study can only be provided to external parties under the conditions of the cooperation contract of this research project and after written approval by the sickness fund. For assistance in obtaining access to the data, please contact wido@wido.bv.aok.de.

## Supplementary Materials

The following are available online at https://www.mdpi.com/article/10.3390/jcm10112500/s1, Table S1: Definition of all variables including diagnosis, procedure and pharmaceutical codes and endpoints. Locked plate fixation—LPF, reverse total shoulder arthroplasty—RTSA. Table S2: Comorbidities, pharmacotherapy und complications during index case depending on sex. Table S3: Results of multivariable Cox regression models for different endpoints presented in Figure 3. Confidence interval—CI, follow-up—FU, intra-hospital—IH, length of hospitalization—LOS, locked plate fixation—LPF, reverse total shoulder arthroplasty—RTSA.
