# Supplementary material for: Male Sex Is Associated with Higher Mortality and Increased Risk for Complications after Surgical Treatment of Proximal Humeral Fractures
Source: J Clin Med. 2021 Jun 5;10(11):2500. doi: 10.3390/jcm10112500 (PMC8201359; doi:10.3390/jcm10112500)
Supplement: Supplementary file 1 [file jcm-10-02500-s001.zip › jcm-1238655-supplementary.pdf]

Supplementary table S1: Definition of all variables including diagnosis, procedure and pharmaceutical codes and endpoints. locked plate fixation – LPF, reverse total shoulder arthroplasty – RTSA.

Variable	Classification	Code
Proximal humeral fracture	ICD	S42.2
LPF	OPS	5-794.21, 5-794.k1
RTSA	OPS	5-824.21
**Comorbidities at baseline:**		
Alcohol abuses	ICD	F10, K70, G31.2, T51.0, T51.9, K29.2, K86.0, I42.6, K85.2, G62.1, G72.1, E24.4
Atherosclerosis	ICD	I70
Atrial fibrillation and flutter	ICD	I48
Bone tumor/metastasis	ICD	C40.0, C79.5, C79.86, C79.9
Cancer	ICD	C
Chronic kidney disease	ICD	N18, N19
Chronic polyarthritis	ICD	M05, M06
Congestive heart failure	ICD	I50
Coronary heart disease	ICD	I25
Dementia	ICD	F00, F01, F02, F051 , G30, G31.1
Diabetes mellitus	ICD	E10-14
Hypertension	ICD	I10-I15
Infection	ICD	T84.5, T84.6, T84.7, M86.01, M86.11, M86.21, M86.31, M86.41, M86.51, M86.61, M86.81, M86.91
Nicotine abuses	ICD	F17
Obesity	ICD	E66
Osteoporosis	ICD	M80-M85
Parkinson	ICD	G20
Polytrauma	ICD, OPS, DRG	ICD: T07, OPS: 5-982, or coded DRG started with "W"
Previous stroke	ICD	I60-I69
**General complications:**		
Acute liver failure	ICD	K72.0, K72.7, K72.9
Acute myocardial infarction	ICD	I21, I22
Acute renal failure	ICD	N17
Acute respiratory distress syndrome	ICD	J80
Cardiac arrest	ICD	I46
Deep vein thrombosis	ICD	I80.1, I80.2, I82.2, I82.3
Pulmonary embolism	ICD	I26
Sepsis	ICD	R65.0, R65.1, R65.9, R57.2 (since 2011), A41, A40, B37.7
Stroke	ICD	I60-I64
Ischemic stroke	ICD	Î63-I64
Resuscitation	OPS	8-77
Thrombo-embolic event		Deep vein thrombosis, pulmonary embolism, ischemic stroke
**Surgical complications:**		
Upper limb amputation, ipsilateral (shoulder or upper arm)	OPS	5-862.1, 5-862.2

Delayed union (LPF only; if coded within six months)	OPS	5-782.11, 5-782.21, 5-782.31, 5-782.41, 5-782.51, 5-782.61, 5-782.a1, 5-782.b1, 5-781.a1, 5-784.01, 5-784.11, 5-784.21, 5-784.31, 5-784.41, 5-784.71, 5-784.81, 5-784.b1
Infection	OPS	5-780.41, 5-780.51, 5-780.61, 5-780.71, 5-780.81, 5-780.91, 5-800.20, 5-800.30, 5-800.a0, 5-800.b0, 5-810.10, 5-810.70, 5-810.80, 8-989, 8-989.0, 8-989.1, 8-989.2, 8-989.3, 8-989.4, 8-989.5, 8-989.6
Infection, resistant	OPS	8-987, 8-987.0, 8-987.00, 8-987.01, 8-987.02, 8-987.03, 8-987.1, 8-987.10, 8-987.11, 8-987.12, 8-987.13
Joint damage / cartilage damage (LPF only)	OPS	5-782.11, 5-782.21, 5-782.31, 5-782.41, 5-782.51, 5-782.61, 5-782.a1, 5-782.b1, 5-781.a1, 5-784.01, 5-784.11, 5-784.21, 5-784.31, 5-784.41, 5-784.71, 5-784.81, 5-784.b1
Luxation	OPS	8-201.0
Malunion (LPF only)	OPS	5-781.01, 5-781.11, 5-781.21, 5-781.31, 5-781.41, 5-781.51, 5-781.61, 5-781.81, 5-781.91
Nerve injury	OPS	5-040.1, 5-040.2, 5-040.3, 5-041.1, 5-041.2, 5-041.3, 5-044.1, 5-044.2, 5-044.3, 5-045.1, 5-045.2, 5-045.3, 5-046.1, 5-046.2, 5-046.3, 5-047.1, 5-047.2, 5-047.3, 5-048.1, 5-048.2, 5-048.3, 5-049.1, 5-049.2, 5-049.3, 5-04b.1, 5-04b.2, 5-04b.3, 5-050.1, 5-050.2, 5-050.3, 5-051.1, 5-051.2, 5-051.3, 5-052.1, 5-052.2, 5-052.3, 5-053.1, 5-053.2, 5-053.3, 5-054.1, 5-054.2, 5-054.3, 5-055.1, 5-055.2, 5-055.3, 5-056.1, 5-056.2, 5-056.3, 5-057.1, 5-057.2, 5-057.3
Non-union / Pseudoarthrosis (LPF only; if coded after six months)	OPS	5-782.11, 5-782.21, 5-782.31, 5-782.41, 5-782.51, 5-782.61, 5-782.a1, 5- 782.b1, 5-781.a1, 5-784.01, 5-784.11, 5-784.21, 5-784.31, 5-784.41, 5-784.71, 5-784.81, 5-784.b1
Osteonecrosis (LPF only)	ICD	M87.21, M87.22, M87.32, M87.82, M87.92
Postoperative stiffness, Adhesive capsulitis, Frozen shoulder	OPS	5-800.60, 5-810.20, 5-810.90, 5-800.c0
Vascular injury	OPS	5-388.11, 5-388.12, 5-395.11, 5-395.12, 5-397.11, 5-397.12
Secondary surgery, open (LPF)	OPS	5-780.01, 5-780.11, 5-780.21, 5-780.31, 5-780.61, 5-780.x1, 5-782.11, 5-782.21, 5-782.31, 5-782.41, 5-782.51, 5-782.62, 5-782.72, 5-782.82, 5-782.92, 5-782.a1, 5-784.01, 5-784.11, 5-784.21, 5-784.31, 5-784.41, 5-784.51, 5-784.61, 5-784.71, 5-784.81, 5-784.a1, 5-784.b1, 5-785.01, 5-785.11, 5-785.21, 5-785.31, 5-785.41, 5-785.51, 5-785.61, 5-785.71, 5-789.b1, 5-789.c1, 5-794.01, 5-794.11, 5-794.21, 5-794.41, 5-794.71, 5-794.81, 5-794.k1, 5-800.10, 5-800.30, 5-800.40, 5-800.50, 5-800.70, 5-800.80, 5-800.90, 5-800.x0, 5-801.00, 5-801.30, 5-801.40, 5-801.b0, 5-801.c0, 5-801.g0, 5-801.h0, 5-801.k0, 5-801.m0, 5-801.n0, 5-801.p0, 5-805.6, 5-805.7, 5-805.8, 5-805.9, 5-805.a, 5-850.01, 5-850.11, 5-850.21, 5-850.31, 5-850.41, 5-850.51, 5-850.61, 5-850.71, 5-850.81, 5-850.91, 5-850.a1, 5-850.b1, 5-850.c1, 5-850.d1, 5-850.e1, 5-850.f1, 5-850.g1, 5-850.h1, 5-850.j1, 5-850.x1, 5-851.11, 5-851.21, 5-852.01, 5-852.11, 5-853.01, 5-853.11, 5-855.01, 5-855.11, 5-855.21, 5-855.51, 5-855.61, 5-855.71, 5-855.81, 5-855.91, 5-855.a1, 5-859.01, 5-859.11, 5-862.1, 5-862.2, 5-892.06, 5-892.07, 5-892.16, 5-892.17, 5-896.06, 5-896.16, 5-896.26, 8-115.0
Secondary surgery, open (RTSA)	OPS	5-780.01, 5-780.11, 5-780.21, 5-780.31, 5-780.61, 5-780.x1, 5-782.11, 5-782.21, 5-782.31, 5-782.41, 5-782.51, 5-782.62, 5-782.72, 5-782.82, 5-782.92, 5-782.a1, 5-785.01, 5-785.11, 5-785.21, 5-785.31, 5-785.41, 5-785.51, 5-785.61, 5-785.71, 5-789.b1, 5-791.02, 5-791.12, 5-791.22, 5-792.k2, 5-792.02, 5-792.12, 5-792.22, 5-792.k2, 5-800.10, 5-800.30, 5-800.40, 5-800.50, 5-800.70, 5-800.80, 5-800.90, 5-800.x0, 5-850.01, 5-850.11, 5-850.21, 5-850.31, 5-850.41, 5-850.51, 5-850.61, 5-850.71, 5-850.81, 5-850.91, 5-850.a1, 5-850.b1, 5-850.c1, 5-850.d1, 5-850.e1, 5-850.f1, 5-850.g1, 5-850.h1, 5-850.j1,

		5-850.x1, 5-859.01, 5-859.11, 5-862.1, 5-862.2, 5-892.06, 5-892.07, 5-892.16, 5-892.17 5-896.06, 5-896.16, 5-896.26
Secondary arthroscopy (LPF)	OPS	5-782.b1, 5-784.c1, 5-784.d1, 5-784.e1, 5-784.f1, 5-810.00, 5-810.10, 5-810.20, 5-810.40, 5-810.50, 5-810.70, 5-810.80, 5-810.90, 5-811.20, 5-811.30, 5-811.40 (since 2011), 5-812.00, 5-812.30, 5-812.90, 5-812.a0, 5-812.e0, 5-812.f0, 5-812.g0, 5-812.h0, 5-812.k0 (since 2011), 5-812.m0, 5-814.0, 5-814.1, 5-814.2, 5-814.3, 5-814.4, 5-812.40, 5-814.5, 5-814.6, 5-814.7 (since 2011), 5-814.8 (since 2011), 5-814.9 (since 2011), 5-814.b (since 2011), 5-814.c, 5-814.d, 5-814.e, 5-819.00, 5-819.20 (since 2011)
Secondary arthroscopy (RTSA)	OPS	5-782.b1, 5-810.00, 5-810.10, 5-810.20, 5-810.40, 5-810.50, 5-810.70, 5-810.80, 5-810.90, 5-811.20, 5-811.30, 5-811.40 (since 2011), 5-814.b (since 2011), 5-814.c, 5-814.d, 5-814.e, 5-819.00, 5-819.10 (since 2011), 5-819.20 (since 2011)
Revision (LPF)	OPS	5-794.21, 5-794.k1, 5-824.21, 5-825.00 (since 2011), 5-825.10, 5-825.1 (2010-2012), 5-825.11, 5-825.1 (2010-2012), 5-825.12, 5-825.1 (2010-2012), 5-825.20, 5-825.21, 5-825.k, 5-825.k0, 5-825.k1, 5-825.kx, 5-825.1 (only 2010-2012), 5-78a.01, 5-78a.11, 5-78a.21,5-78a.31, 5-78a.k1, 5-785.01, 5-785.11, 5-789.31, 5-794.11, 5-794.31, 5-794.41, 5-794.71, 5-794.81, 5-824.015-793.01, 5-793.11, 5-793.21, 5-793.31, 5-793.41, 5-793.51, 5-793.a1, 5-793.b1, 5-793.h1, 5-793.k1
Revision (RTSA)	OPS	5-824.21, 5-825.00 (since 2011), 5-825.21, 5-825.8, 5-825.k, 5-825.k0, 5-825.k1, 5-825.kx, 5-825.1 (only 2010-2012), 5-787.01, 5-787.11, 5-787.21, 5-787.31, 5-787.k1, 5-810.40, 5-78a.01, 5-78a.11, 5-78a.21, 5-78a.k1, 5-785.01, 5-785.11, 5-789.31
Resection arthroplasty (for spacer placement)	OPS	5-829.3
Secondary arthroplasty (LPF only)	OPS	5-824.0, 5-824.20, 5-824.21
Arthrolysis (LPF only)	OPS	5-800.60, 5-810.20, 5-810.90
Decompression of subacromial space (LPF only)	OPS	5-814.3
Debridement (LPF only)	OPS	5-819.10 (since 2011)
Previous surgery (shoulder)	OPS	5-040.1, 5-041.1, 5-044.1, 5-045.1, 5-046.1, 5-047.1, 5-048.1, 5-049.1, 5-04b.1, 5-050.1, 5-051.1, 5-052.1, 5-053.1, 5-054.1, 5-055.1, 5-056.1, 5-057.1, 5-388.11, 5-388.12, 5-395.11, 5-395.12, 5-397.11, 5-397.12, 5-780.01, 5-780.11, 5-780.21, 5-780.31, 5-780.41, 5-780.51, 5-780.61, 5-780.71, 5-780.81, 5-780.91, 5-780.x1, 5-781.01, 5-781.11, 5-781.21, 5-781.31, 5-781.41, 5-781.51, 5-781.61, 5-781.71, 5-781.81, 5-781.91, 5-781.a1, 5-781.x1, 5-782.11, 5-782.21, 5-782.31, 5-782.41, 5-782.51, 5-782.62, 5-782.72, 5-782.82, 5-782.92, 5-782.a1, 5-782.b1, 5-782.x1, 5-784.01, 5-784.11, 5-784.21, 5-784.31, 5-784.41, 5-784.51, 5-784.61, 5-784.71, 5-784.81, 5-784.92, 5-784.a1, 5-784.b1, 5-784.c1, 5-784.d1, 5-784.e1, 5-784.f1, 5-784.x1, 5-785.01, 5-785.11, 5-785.21, 5-785.31, 5-785.41, 5-785.51, 5-785.61, 5-785.71, 5-787.01, 5-787.11, 5-787.21, 5-787.31, 5-787.41, 5-787.51, 5-787.61, 5-787.71, 5-787.81, 5-787.91, 5-787.c1, 5-787.g1, 5-787.k1, 5-787.m1, 5-787.n1, 5-787.x1, 5-789.01, 5-789.31, 5-789.41, 5-789.b1, 5-789.c1, 5-789.x1, 5-78a.01, 5-78a.11, 5-78a.21, 5-78a.31, 5-78a.41, 5-78a.51, 5-78a.61, 5-78a.71, 5-78a.81, 5-78a.91, 5-78a.c1, 5-78a.g1, 5-78a.k1, 5-78a.m1, 5-78a.n1, 5-78a.x1, 5-791.02, 5-791.12, 5-791.22, 5-791.32, 5-791.42, 5-791.52, 5-791.62, 5-791.72, 5-791.82, 5-791.92, 5-791.c2, 5-791.d2, 5-791.g2, 5-791.h2, 5-791.k2, 5-791.m2, 5-791.n2, 5-791.x2, 5-792.02, 5-792.12, 5-792.22, 5-792.32, 5-792.42, 5-792.52, 5-792.62, 5-792.72, 5-792.82, 5-792.92, 5-792.g2, 5-792.h2, 5-792.k2, 5-792.m2,

		5-792.n2, 5-792.x2, 5-793.01, 5-793.11, 5-793.21, 5-793.31, 5-793.41, 5-793.51, 5-793.61, 5-793.71, 5-793.81, 5-793.91, 5-793.a1, 5-793.b1, 5-793.c1, 5-793.g1, 5-793.h1, 5-793.k1, 5-793.m1, 5-793.n1, 5-793.x1, 5-794.01, 5-794.11, 5-794.21, 5-794.31, 5-794.41, 5-794.51, 5-794.61, 5-794.71, 5-794.81, 5-794.a1, 5-794.b1, 5-794.c1, 5-794.g1, 5-794.h1, 5-794.k1, 5-794.m1, 5-794.n1, 5-794.x1, 5-79a.00, 5-79a.10, 5-79a.60, 5-79a.70, 5-79a.80, 5-79a.e0, 5-79a.g0, 5-79a.x0, 5-79b.00, 5-79b.10, 5-79b.20, 5-79b.60, 5-79b.70, 5-79b.80, 5-79b.e0, 5-79b.g0, 5-79b.h0, 5-79b.x0, 5-800.00, 5-800.10, 5-800.20, 5-800.30, 5-800.40, 5-800.50, 5-800.60, 5-800.70, 5-800.80, 5-800.90, 5-800.a0, 5-800.b0, 5-800.c0, 5-800.x0, 5-801.00, 5-801.30, 5-801.40, 5-801.b0, 5-801.c0, 5-801.g0, 5-801.h0, 5-801.k0, 5-801.m0, 5-801.n0, 5-801.p0, 5-801.x0, 5-805.0, 5-805.1, 5-805.2, 5-805.3, 5-805.4, 5-805.5, 5-805.6, 5-805.7, 5-805.8, 5-805.9, 5-805.a, 5-805.b, 5-805.x, 5-805.y, 5-808.4, 5-809.00, 5-809.10, 5-809.20, 5-809.40, 5-809.x0, 5-810.00, 5-810.10, 5-810.20, 5-810.30, 5-810.40, 5-810.50, 5-810.60, 5-810.70, 5-810.80, 5-810.90, 5-810.x0, 5-811.20, 5-811.30, 5-811.40, 5-811.x0, 5-812.00, 5-812.30, 5-812.90, 5-812.a0, 5-812.e0, 5-812.f0, 5-812.g0, 5-812.h0, 5-812.k0, 5-812.m0, 5-812.x0, 5-814, 5-819.00, 5-819.10, 5-819.20, 5-819.x0, 5-824.0, 5-824.20, 5-824.21, 5-825.00, 5-825.1, 5-825.2, 5-825.20, 5-825.21, 5-825.2x, 5-825.7, 5-825.8, 5-825.h, 5-825.j, 5-825.k, 5-828.11, 5-828.41, 5-828.61, 5-829.j, 5-829.k, 5-850.01, 5-850.11, 5-850.21, 5-850.31, 5-850.41, 5-850.51, 5-850.61, 5-850.71, 5-850.81, 5-850.91, 5-850.a1, 5-850.b1, 5-850.c1, 5-850.d1, 5-850.e1, 5-850.f1, 5-850.g1, 5-850.h1, 5-850.j1, 5-850.x1, 5-851.01, 5-851.11, 5-851.21, 5-851.31, 5-851.41, 5-851.51, 5-851.61, 5-851.71, 5-851.81, 5-851.91, 5-851.a1, 5-851.b1, 5-851.c1, 5-851.d1, 5-851.x1, 5-852.01, 5-852.11, 5-852.21, 5-852.31, 5-852.41, 5-852.51, 5-852.61, 5-852.71, 5-852.81, 5-852.91, 5-852.a1, 5-852.b1, 5-852.c1, 5-852.d1, 5-852.h1, 5-852.j1, 5-852.x1, 5-853.01, 5-853.11, 5-853.21, 5-853.31, 5-853.41, 5-853.51, 5-853.61, 5-853.71, 5-853.81, 5-853.91, 5-853.x1, 5-854.01, 5-854.11, 5-854.21, 5-854.31, 5-854.41, 5-854.51, 5-854.61, 5-854.71, 5-854.81, 5-854.91, 5-854.a1, 5-854.x1, 5-855.01, 5-855.11, 5-855.21, 5-855.31, 5-855.41, 5-855.51, 5-855.61, 5-855.71, 5-855.81, 5-855.91, 5-855.a1, 5-855.b1, 5-855.x1, 5-856.01, 5-856.11, 5-856.21, 5-856.31, 5-856.41, 5-856.51, 5-856.61, 5-856.71, 5-856.81, 5-856.91, 5-856.a1, 5-856.x1, 5-859.01, 5-859.11, 5-859.21, 5-859.31, 5-859.x1, 5-862.0, 5-862.1, 5-862.2, 5-86a.3, 5-896.06, 5-896.16, 5-896.26, 5-896.x6, 5-983, 8-115.0
**Pharmaceutical therapy:**		
Any anticoagulant	ATC	B01AA, B01AB, B01AC, B01AE, B01AF, B01AX
Vitamin D or calcium	ATC	A11CC
Bisphosphonates	ATC	M05BA, M05BB
Any osteoporosis pharmacotherapy	ATC	Vitamin D/Calcium or Bisphosphonates
**Classification of complications and long-term endpoints/events:**		
Major adverse event		resuscitation, cardiac arrest, myocardial infarction, stroke, acute renal failure, acute liver failure, acute respiratory distressed syndrome, sepsis or death from any case
Surgical complications		Adhesive capsulitis, arthrolysis, debridement, decompression of subacromial space, frozen shoulder, infection, infection with antibiotic-resistant germs, joint damage/cartilage damage, luxation, delayed union, non-union/ pseudoarthrosis, malunion, nerve injury, vascular injury, osteonecrosis, postoperative stiffness, secondary

		arthroplasty, secondary arthroscopy, secondary surgery (open) including revision surgery, upper limb amputation
Any secondary surgery (shoulder, ipsilateral)		All surgical intervention on the same side coded during follow-up, i.e. all secondary interventions coded with an OPS from this list beginning with "5-"
Minor outpatient complications	ICD	G56.1, G56.2, G56.3, I80.80, I80.81, M00.01, M00.11, M00.21, M00.81, M00.91, M24.21, M24.41, M24.31, M24.51, M24.61, M25.11, M25.21, M25.31, M25.41, M25.51, M25.61, M25.71, M61.01, M62.21, M62.41, M62.51, M62.61, M65.81, M65.91, M75.0, M75.1, M75.2, M75.4, M75.5, M84.31, M86.01, M86.11, M86.21, M86.31, M86.41, M86.51, M86.61, M86.81, M86.91, M89.51, M96.6, S41.86, S43.0, S44, S45.0, S45.1, S46.0, S55.0, S55.1, T79.60, T81.4, T84.5, T84.6, T84.7, T84.00 (only RTSA), Only LPF: M13.11, M13.81, M13.91, M19.11, M24.01, M24.11, M62.22, M84.21, M87.21, M87.22, M87.32, M87.82, M87.92, M89.51

Supplementary table 2: Comorbidities, pharmacotherapy und complications during index case depending on sex.

	**All patients N= 53,971 (100%)**	**Female sex N=45,707 (84.7%)**	**Male sex N=8,264 (15.4%)**	**p value**
Median age – years (IQR)	79 (10)	79 (10)	76 (11)	<.001
Age ≥ 80 years – n (%)	24,869 (46.1%)	21,950 (48.0%)	2,919 (35.3%)	<.001
Reverse total shoulder arthroplasty – n (%)	13,552 (25.1%)	11,793 (25.8%)	1,759 (21.3%)	<.001
Osteoporosis – n (%)	19,677 (36.5%)	18,383 (40.2%)	1,294 (15.7%)	<.001
Cancer – n (%)	11,877 (22.0%)	9,502 (20.8%)	2,375 (28.7%)	<.001
Diabetes mellitus – n (%)	21,640 (40.1%)	18,178 (39.8%)	3,462 (41.9%)	<.001
Dementia – n (%)	6,781 (12.6%)	5,739 (12.6%)	1,042 (12.6%)	0.894
Chronic polyarthritis – n (%)	3,305 (6.1%)	3,026 (6.6%)	279 (3.4%)	<.001
Obesity – n (%)	15,360 (28.5%)	13,261 (29.0%)	2,099 (25.4%)	<.001
Nicotin abuses – n (%)	3,090 (5.7%)	1,983 (4,3%)	1,107 (13.4%)	<.001
Parkinson – n (%)	2,152 (4.0%)	1,703 (3.7%)	449 (5.4%)	<.001
Previous surgery (shoulder) – n (%)	230 (0.4%)	186 (0.4%)	44 (0.5%)	0.107
Rotator cuff rupture – n (%)	3,931 (7.3%)	3,363 (7.4%)	568 (6.9%)	0.119
Alcohol abuses – n (%)	2,921 (5.4%)	1,415 (3.1%)	1,506 (18.2%)	<.001
Previous Stroke – n (%)	13,575 (25.2%)	11,180 (24.5%)	2,395 (29.0%)	<.001
Omarthrosis– n (%)	1,250 (2.3%)	1,082 (2.4%)	168 (2.0%)	0.063
Frozen shoulder – n (%)	2,430 (4.5%)	2,140 (4.7%)	290 (3.5%)	<.001
Atrial fibrillation and flutter – n (%)	10,298 (19.1%)	8,414 (18.4%)	1,884 (22.8%)	<.001
Congestive heart failure – n (%)	16,277 (30.2%)	13,764 (30.1%)	2,513 (30.4%)	0.590
Coronary heart disease – n (%)	16,588 (30.7%)	13,551 (29.7%)	3,037 (36.8%)	<.001
Hypertension – n (%)	47,475 (88.0%)	40,479 (88.6%)	6,996 (84.7%)	<.001
Atherosclerosis – n (%)	9,172 (17.0%)	7,384 (16.2%)	1,788 (21.6%)	<.001
Chronic kidney disease – n (%)	15,152 (28.1%)	12,612 (27.6%)	2,540 (30.7%)	<.001
Median Charlson comorbidity index – (IQR)	3 (3)	3 (3)	3 (4)	
Charlson comorbidity index – n (%)				<.001
0	9,104 (16.9%)	7,944 (17.4%)	1,160 (14.0%)	
1	7,721 (14.3%)	6,701 (14.7%)	1,020 (12.3%)	
2	9,288 (17.2%)	8,026 (17.6%)	1,262 (15.3%)	
3	8,268 (15.3%)	7,054 (15.4%)	1,214 (14.7%)	
4	6,752 (12.5%)	5,656 (12.4%)	1,096 (13.3%)	
5	4,686 (8.7%)	3,857 (8.4%)	829 (10.0%)	
>5	8,152 (15.1%)	6,469 (14.2%)	1,683 (20.4%)	
**Medication at admission – n (%):**				
Any anticoagulant	15,375 (28.5%)	12,550 (27.5%)	2,825 (34.2%)	<.001

Vitamin D or Calcium	2,357 (4.4%)	2,109 (4.6%)	248 (3.0%)	<.001
Bisphosphonates	3,781 (7.0%)	3,621 (7.9%)	160 (1.9%)	<.001
Any osteoporosis pharmacotherapy	5,543 (10.3%)	51,162 (11.3%)	381 (4.6%)	<.001
**Index case:**				
Mean duration of hospitalization – days (+/- SD)	15.8 (±12.0)	15.8 (±11.7)	16.3 (±13.3)	<.001
Major medical complications	4,905 (9.1%)	3,866 (8.5%)	1,039 (12.6%)	<.001
Surgical complications	4,248 (7.9%)	3,340 (7.3%)	908 (11.0%)	<.001

Supplementary table S3: Results of multivariable Cox regression models for different endpoints presented in figure 3. confidence interval – CI, follow-up – FU, intra-hospital – IH, length of hospitalization – LOS, locked plate fixation – LPF, reverse total shoulder arthroplasty – RTSA.

Variable	Hazard ratio	95% CI	P value
**Overall survival**			
Male vs female sex after LPF	1.67	1.60 – 1.75	<.001
Male vs female sex after RTSA	1.53	1.40 – 1.68	<.001
Age at index surgery	1.09	1.08 – 1.09	<.001
Diabetes mellitus	1.25	1.21 – 1.29	<.001
Dementia	1.84	1.77 – 1.91	<.001
Chronic polyarthritis	1.09	1.02 – 1.16	0.009
Obesity	0.88	0.85 – 0.91	<.001
Nicotine abuses	1.60	1.50 – 1.71	<.001
Parkinson	1.31	1.23 – 1.40	<.001
Rotator cuff rupture	0.87	0.82 – 0.93	<.001
Alcohol abuses	1.71	1.60 – 1.82	<.001
Previous stroke	1.05	1.01 – 1.09	0.006
Omarthrosis	0.86	0.76 – 0.97	0.014
Frozen shoulder	0.81	0.75 – 0.88	<.001
Previous surgery (shoulder)	0.91	0.70 – 1.20	0.512
Atrial fibrillation and flutter	1.31	1.26 – 1.37	<.001
Congestive heart failure	1.30	1.26 – 1.34	<.001
Coronary heart disease	1.00	0.96 – 1.03	0.776
Hypertension	0.98	0.92 – 1.03	0.388
Atherosclerosis	1.01	0.97 – 1.05	0.693
Chronic kidney disease	1.24	1.20 – 1.28	<.001
Osteoporosis	1.02	0.99 – 1.06	0.179
Cancer	1.14	1.10 – 1.18	<.001
Any anticoagulant	1.08	1.04 – 1.12	<.001
Any osteoporosis pharmacotherapy	1.06	1.01 – 1.12	0.021
Year of index surgery	0.99	0.98 – 1.00	0.006
LOS index case	1.01	1.01 – 1.01	<.001
Surgical compl. IH index case	1.11	1.05 – 1.17	<.001
General compl. IH index case	1.43	1.37 – 1.50	<.001
**Major adverse event**			
Male vs female sex after LPF	1.58	1.51 – 1.64	<.001
Male vs female sex after RTSA	1.44	1.33 – 1.56	<.001
Age at index surgery	1.06	1.06 – 1.07	<.001

Diabetes mellitus	1.28	1.24 – 1.31	<.001
Dementia	1.60	1.55 – 1.66	<.001
Chronic polyarthritis	1.11	1.05 – 1.17	<.001
Obesity	0.95	0.92 – 0.98	0.001
Nicotine abuses	1.44	1.36 – 1.53	<.001
Parkinson	1.29	1.22 – 1.37	<.001
Rotator cuff rupture	0.93	0.88 – 0.98	0.008
Alcohol abuses	1.58	1.50 – 1.68	<.001
Previous stroke	1.11	1.07 – 1.14	<.001
Omarthrosis	0.87	0.78 – 0.96	0.006
Frozen shoulder	0.86	0.80 – 0.92	<.001
Previous surgery (shoulder)	1.03	0.83 – 1.27	0.819
Atrial fibrillation and flutter	1.27	1.23 – 1.32	<.001
Congestive heart failure	1.25	1.21 – 1.29	<.001
Coronary heart disease	1.04	1.01 – 1.07	0.015
Hypertension	1.04	0.99 – 1.09	0.170
Atherosclerosis	1.02	0.99 – 1.06	0.265
Chronic kidney disease	1.31	1.27 – 1.36	<.001
Osteoporosis	1.01	0.98 – 1.04	0.468
Cancer	1.09	1.05 – 1.12	<.001
Any anticoagulant	1.08	1.04 – 1.11	<.001
Any osteoporosis pharmacotherapy	1.06	1.02 – 1.12	0.006
Year of index surgery	1.00	0.99 – 1.01	0.743
LOS index case	1.01	1.01 – 1.01	<.001
Surgical compl. IH index case	1.09	1.04 – 1.15	<.001
General compl. IH index case	1.45	1.39 – 1.52	<.001
**Thromboembolic event (or death)**			
Male vs female sex after LPF	1.53	1.47 – 1.60	<.001
Male vs female sex after RTSA	1.42	1.31 – 1.55	<.001
Age at index surgery	1.07	1.07 – 1.08	<.001
Diabetes mellitus	1.24	1.20 – 1.27	<.001
Dementia	1.70	1.63 – 1.76	<.001
Chronic polyarthritis	1.11	1.05 – 1.18	<.001
Obesity	0.92	0.89 – 0.95	<.001
Nicotine abuses	1.51	1.42 – 1.60	<.001
Parkinson	1.27	1.20 – 1.35	<.001
Rotator cuff rupture	0.91	0.86 – 0.97	0.002
Alcohol abuses	1.57	1.47 – 1.67	<.001
Previous stroke	1.10	1.07 – 1.14	<.001
Omarthrosis	0.87	0.78 – 0.97	0.010
Frozen shoulder	0.82	0.76 – 0.88	<.001
Previous surgery (shoulder)	0.93	0.73 – 1.18	0.530
Atrial fibrillation and flutter	1.25	1.21 – 1.30	<.001
Congestive heart failure	1.24	1.20 – 1.28	<.001
Coronary heart disease	1.00	0.97 – 1.03	0.925
Hypertension	0.98	0.93 – 1.03	0.369
Atherosclerosis	1.01	0.97 -. 1.05	0.602
Chronic kidney disease	1.23	1.19 – 1.27	<.001
Osteoporosis	1.03	1.00 – 1.07	0.039
Cancer	1.12	1.08 – 1.16	<.001
Any anticoagulant	1.07	1.04 – 1.11	<.001
Any osteoporosis pharmacotherapy	1.07	1.02 – 1.12	0.011

Year of index surgery	0.99	0.98 – 1.00	0.003
LOS index case	1.01	1.01 – 1.01	<.001
Surgical compl. IH index case	1.09	1.04 – 1.15	<.001
General compl. IH index case	1.40	1.34 – 1.46	<.001
**Surgical complications only during FU (after discharge)**			
Male vs female sex after LPF	0.98	0.90 – 1.06	1.000
Male vs female sex after RTSA	1.86	1.56 – 2.22	<.001
Age at index surgery	0.97	0.96 – 0.97	<.001
Diabetes mellitus	1.03	0.97 – 1.09	0.316
Dementia	0.76	0.68 – 0.84	<.001
Chronic polyarthritis	1.16	1.04 – 1.28	0.006
Obesity	1.17	1.10 – 1.26	<.001
Nicotine abuses	1.06	0.96 – 1.19	0.258
Parkinson	1.08	0.93 – 1.24	0.316
Rotator cuff rupture	1.10	1.00 – 1.22	0.057
Alcohol abuses	1.26	1.13 – 1.41	<.001
Previous stroke	1.01	0.94 – 1.08	0.786
Omarthrosis	1.07	0.90 – 1.27	0.466
Frozen shoulder	1.17	1.04 – 1.31	0.009
Previous surgery (shoulder)	1.22	0.84 – 1.75	0.296
Atrial fibrillation and flutter	0.93	0.85 – 1.01	0.066
Congestive heart failure	1.04	0.97 – 1.11	0.247
Coronary heart disease	0.98	0.92 – 1.05	0.560
Hypertension	1.12	1.02 – 1.22	0.014
Atherosclerosis	1.03	0.95 – 1.11	0.485
Chronic kidney disease	1.07	1.00 – 1.14	0.059
Osteoporosis	1.12	1.05 – 1.19	<.001
Cancer	0.96	0.89 – 1.02	0.172
Any anticoagulant	1.05	0.98 – 1.12	0.198
Any osteoporosis pharmacotherapy	1.03	0.94 – 1.13	0.553
Year of index surgery	0.99	0.98 – 1.00	0.138
LOS index case	1.00	0.99 – 1.00	<.001
Surgical compl. IH index case	1.63	1.50 – 1.78	<.001
General compl. IH index case	1.00	0.89 – 1.11	0.934
**Surgical complications incl. index case**			
Male vs female sex after LPF	1.14	1.07 – 1.22	<.001
Male vs female sex after RTSA	1.70	1.51 – 1.92	<.001
Age at index surgery	0.99	0.98 – 0.99	<.001
Diabetes mellitus	1.01	0.97 – 1.06	0.575
Dementia	0.91	0.85 – 0.98	0.009
Chronic polyarthritis	1.04	0.96 – 1.13	0.354
Obesity	1.11	1.06 – 1.16	<.001
Nicotine abuses	1.05	0.96 – 1.14	0.297
Parkinson	1.08	0.97 – 1.19	0.154
Rotator cuff rupture	1.19	1.10 – 1.28	<.001
Alcohol abuses	1.33	1.22 – 1.44	<.001
Previous stroke	0.96	0.91 – 1.01	0.119
Omarthrosis	1.04	0.91 – 1.18	0.604
Frozen shoulder	1.11	1.01 – 1.22	0.030
Previous surgery (shoulder)	1.70	1.33 – 2.16	<.001
Atrial fibrillation and flutter	1.00	0.94 – 1.06	0.980
Congestive heart failure	1.04	0.99 – 1.10	0.114
Coronary heart disease	0.96	0.91 – 1.01	0.085

Hypertension	1.11	1.04 – 1.19	0.003
Atherosclerosis	1.00	0.95 – 1.06	0.975
Chronic kidney disease	1.09	1.04 – 1.15	<.001
Osteoporosis	1.13	1.08 – 1.19	<.001
Cancer	0.98	0.93 – 1.03	0.327
Any anticoagulant	1.02	0.97 – 1.07	0.485
Any osteoporosis pharmacotherapy	1.03	0.96 – 1.11	0.359
Year of index surgery	0.99	0.98 – 0.99	<.001
LOS index case	1.01	1.01 – 1.02	<.001
General compl. IH index case	1.25	1.15 – 1.34	<.001
**Any secondary surgery (shoulder, ipsilateral)**			
Male vs female sex after LPF	0.99	0.91 – 1.08	1.000
Male vs female sex after RTSA	1.76	1.46 – 2.12	<.001
Age at index surgery	0.96	0.96 – 0.97	<.001
Diabetes mellitus	1.03	0.97 – 1.09	0.368
Dementia	0.71	0.64 – 0.80	<.001
Chronic polyarthritis	1.15	1.03 – 1.28	0.015
Obesity	1.15	1.08 – 1.23	<.001
Nicotine abuses	1.05	0.93 – 1.17	0.442
Parkinson	1.08	0.93 – 1.25	0.320
Rotator cuff rupture	1.10	1.00 – 1.22	0.063
Alcohol abuses	1.22	1.09 – 1.37	<.001
Previous stroke	1.00	0.93 – 1.07	0.984
Omarthrosis	1.06	0.89 – 1.26	0.531
Frozen shoulder	1.15	1.02 – 1.30	0.020
Previous surgery (shoulder)	1.32	0.92 – 1.90	0.136
Atrial fibrillation and flutter	0.92	0.84 – 1.00	0.049
Congestive heart failure	1.04	0.97 – 1.12	0.282
Coronary heart disease	0.98	0.92 – 1.05	0.537
Hypertension	1.11	1.01 – 1.21	0.027
Atherosclerosis	1.05	0.97 – 1.13	0.236
Chronic kidney disease	1.05	0.98 – 1.13	0.152
Osteoporosis	1.12	1.05 – 1.20	<.001
Cancer	0.97	0.91 – 1.04	0.398
Any anticoagulant	1.07	1.00 – 1.15	0.064
Any osteoporosis pharmacotherapy	1.02	0.92 – 1.12	0.720
Year of index surgery	1.00	0.98 – 1.01	0.384
LOS index case	0.99	0.99 – 1.00	<.001
Surgical compl. IH index case	1.61	1.46 – 1.76	<.001
General compl. IH index case	0.98	0.87 – 1.10	0.708
**Minor outpatientcomplications**			
Male vs female sex after LPF	0.91	0.86 – 0.96	0.004
Male vs female sex after RTSA	1.02	0.92 – 1.12	1.000
Age at index surgery	0.97	0.96 – 0.97	<.001
Diabetes mellitus	0.95	0.92 – 0.98	0.003
Dementia	0.66	0.62 – 0.70	<.001
Chronic polyarthritis	1.12	1.05 – 1.19	<.001
Obesity	1.18	1.14 – 1.22	<.001
Nicotine abuses	0.95	0.89 – 1.02	0.173
Parkinson	1.01	0.92 – 1.10	0.862
Rotator cuff rupture	2.19	2.08 – 2.31	<.001
Alcohol abuses	0.88	0.81 – 0.95	<.001

Previous stroke	0.99	0.95 – 1.03	0.644
Omarthrosis	1.48	1.35 – 1.63	<.001
Frozen shoulder	2.66	2.51 – 2.83	<.001
Previous surgery (shoulder)	1.00	0.81 – 1.22	0.965
Atrial fibrillation and flutter	0.88	0.84 – 0.93	<.001
Congestive heart failure	1.04	0.99 – 1.08	0.096
Coronary heart disease	1.06	0.99 – 1.08	0.096
Hypertension	1.13	1.07 – 1.19	<.001
Atherosclerosis	1.14	1.09 – 1.19	<.001
Chronic kidney disease	0.95	0.92 – 0.99	0.022
Osteoporosis	1.15	1.11 – 1.19	<.001
Cancer	1.06	1.02 – 1.10	0.006
Any anticoagulant	1.01	0.97 – 1.05	0.680
Any osteoporosis pharmacotherapy	1.09	1.03 – 1.15	0.002
Year of index surgery	1.01	1.01 – 1.02	<.001
LOS index case	1.00	0.99 – 1.00	<.001
Surgical compl. IH index case	1.30	1.23 – 1.38	<.001
General compl. IH index case	0.90	0.84 – 0.96	0.001
